# When Hormones Attack: A Literature Review of Progesterone-Induced Anaphylaxis

**DOI:** 10.1007/s11882-026-01267-4

**Published:** 2026-03-19

**Authors:** Roxana Silvia Bumbăcea, Denisa-Alexandra Băloiu, Mihaela Ruxandra Vintilă, Maria Lucia Toader, Selda Ali

**Affiliations:** 1 Carol Davila University of Medicine and Pharmacy, Bucharest, 050474 Romania; 2 Allergology Department, Dr. Carol Davila Nephrology Clinical Hospital, Bucharest, 010731 Romania

**Keywords:** Progesterone, Progestogen hypersensitivity, Anaphylaxis, Diagnosis and treatment

## Abstract

**Purpose of Review:**

Progesterone hypersensitivity is a rare and underdiagnosed condition whose incidence is expected to rise due to the increasing use of assisted reproductive technologies and exogenous progesterone exposure. This review aims to summarize the reported cases of endogenous and exogenous progesterone-induced anaphylaxis, focusing on clinical manifestations, diagnostic strategies, and management options.

**Recent Findings:**

A literature search through three data bases identified 25 documented cases of progesterone-induced anaphylaxis. Both endogenous (*n* = 15) and exogenous (*n* = 10) exposures were implicated, with severe reactions including grade 4 (*n* = 12) and grade 5 (*n* = 1) anaphylaxis. Diagnostic evaluation commonly involved skin testing and challenge procedures, although heterogeneity in protocols was evident. Management strategies varied widely: some patients responded to hormonal modulation, while others required oophorectomy or treatment with monoclonal antibodies. Desensitization protocols were successfully implemented in selected cases, including in the context of in vitro fertilization.

**Summary:**

PH should be recognized as a potentially life-threatening condition, particularly relevant for women of reproductive age undergoing assisted reproduction or hormonal therapy. By combining clinical insights with published examples of successful diagnostic and therapeutic strategies, this review aims to support clinicians in recognizing progesterone-induced anaphylaxis and implementing patient-centered management. Increased awareness is essential for early diagnosis and individualized treatment.

## Introduction

Progesterone hypersensitivity (PH) is a rare and likely underdiagnosed condition that primarily affects women of reproductive age. Clinical manifestations are variable, ranging from skin-limited reactions to life-threatening anaphylaxis. Its under-recognition may lead to misattribution of symptoms, resulting in delayed diagnosis, inappropriate treatment, and significant psychological distress for affected patients.

Endogenous progesterone is a natural steroid hormone synthesized from cholesterol. It is structurally defined by a 21-carbon (C-21) skeleton, known as a pregnane nucleus, which consists of a tetracyclic ring system with ketone groups at positions C3 and C20. In clinical settings, exogenous formulations are categorized into natural (bioidentical) progesterone, which is chemically identical to the endogenous hormone but often requires micronization for bioavailability and synthetic progestins. Synthetic variants are structurally modified to enhance stability and half-life; these include C-21 derivatives (pregnanes), such as medroxyprogesterone acetate or megestrol acetate, which retain the pregnane skeleton and 19-nortestosterone derivatives (estranes), which lack the C19 methyl group [1]. Understanding these structural distinctions is crucial for diagnosing hypersensitivity, as the immune system may develop specific antibodies directed against either the unique structural modifications found in synthetic progestins or the shared steroid nucleus itself, thereby influencing the potential for cross-reactivity between different formulations.

Although still poorly recognized by many healthcare professionals, PH was first described in 1964 by Shelley et al. in 1964 [2], who introduced the term *autoimmune progesterone dermatitis* to refer to cyclical muco-cutaneous symptoms occurring during the luteal phase of the menstrual cycle, when endogenous progesterone levels rise. However, little evidence exists to support an autoimmune mechanism [3–5]. In contrast, several studies have demonstrated that mast cells express receptors for progesterone, and can be activated by this hormone, as can basophils. Furthermore, specific IgE antibodies to progesterone have been repeatedly identified [6–15]. In addition, certain clinical patterns documented in the literature are consistent with non-immediate hypersensitivity reactions to progesterone, indicative of a possible T-cell mediated immune response [16–20].

Moreover, it is important to note that *autoimmune progesterone dermatitis* remains a controversial entity, and its very existence has been questioned by some medical professionals [21]. This skepticism stems from the lack of consistent histopathological findings, leading some authors to suggest that such clinical presentations may in fact represent common progesterone-associated dermatoses rather than a distinct entity [22]. These doubts are particularly relevant in cases of cyclical, skin-limited reactions. However, when clinical manifestations escalate to anaphylaxis requiring treatment with epinephrine, the hypothesis of a hypersensitivity reaction becomes difficult to dismiss. It is essential to emphasize the importance of considering progesterone as a potential trigger in cases of idiopathic anaphylaxis, in order to prompt appropriate allergy investigation and potentially uncover an otherwise overlooked etiology [23].

In this context, Foer et al. [24] proposed replacing the term *autoimmune progesterone dermatitis* with *progestogen hypersensitivity*, a broader designation that encompasses not only endogenous reactions but also responses to exogenous progesterone (both natural and synthetic forms). This terminology reflects a wider clinical spectrum, with potential involvement of various organ systems.

In endogenous PH, symptoms are closely linked to fluctuations in the natural levels of the hormone. Although progesterone is continuously present throughout the menstrual cycle, its concentration increases significantly during the luteal phase, after ovulation, and drops at the onset of menstruation. Moreover, the substantial increase in progesterone levels during pregnancy (resulting from placental hormone production) may serve as a trigger for symptom onset [25, 26]. The exact mechanism of immune sensitization to endogenous hormone remains unclear. In some cases, patients had a prior history of exogenous progesterone exposure, most frequently through combined oral contraceptives. However, sensitization has also been documented in individuals without any known history of exogenous hormone use [27–29].

In exogenous PH, the mechanism of sensitization appears to be more thoroughly understood, as it can resemble a classic drug-induced hypersensitivity reaction. Despite being a hormone, exogenous progesterone differs structurally from the endogenous counterpart, potentially allowing the immune system to activate well-known hypersensitivity pathways. However, the pathogenesis of sensitization remains incompletely understood, and significant knowledge gaps persist [30].

Further studies are needed to clarify the underlying immunological mechanisms, improve diagnostic accuracy, and optimize treatment strategies to provide a personalized approach for affected patients. Accordingly, this review aims to analyze the reported cases of progesterone-induced anaphylaxis, addressing both clinical manifestations and patient-specific factors, in order to raise awareness of this under-recognized condition and support improved diagnosis and care. Given the relative rarity of well-documented cases of progesterone-induced anaphylaxis and the heterogeneity of reporting standards, a narrative review was deemed most appropriate. This approach allows for a flexible yet rigorous synthesis of diverse case reports and management strategies, while enabling critical discussion of variability in diagnostic work-up and therapeutic approaches.

As assisted reproductive techniques, such as in vitro fertilization (IVF), become increasingly necessary, driven by the rising incidence of infertility, the number of PH cases is likely to increase [31]. Therefore, improved recognition and appropriate management strategies are essential for optimal patient care.

## Materials and Methods

We conducted a literature search of articles published before 1st June 2025, using PubMed, Web of Science, and Scopus databases. No start date was applied, as this condition has a longstanding presence in the literature and a longitudinal overview of clinical presentations and allergy approaches over time was considered valuable. Our initial objective was to identify all studies related to PH. We used the following Medical Subject Headings (MeSH) and keywords: “progesterone”, “progestogen”, “hypersensitivity”, “allergy”, and “anaphylaxis”. The search was limited to articles written in English and focused on case reports/ case series. Duplicate records were removed using Rayyan QCRI [32], a web-based tool for managing literature reviews. We adopted a comprehensive and structured approach to evidence synthesis, as the allergy work-up and therapeutic management vary considerably across cases, and a detailed presentation of the data was deemed valuable. Although this is a narrative review, predefined inclusion and exclusion criteria were applied to ensure the relevance and consistency of the selected publications. After screening the identified records, we included only those reporting cases fulfilling the established criteria for progesterone-induced anaphylaxis, which were subsequently analyzed in detail. All authors agreed on the final version of the inclusion and exclusion criteria. The literature search was carried out independently by two authors, and disagreements were resolved by consensus.

For exogenous progesterone anaphylaxis, the inclusion criteria required the presence of clinical manifestations meeting the World Allergy Organization (WAO) 2020 diagnostic criteria for anaphylaxis [33] occurring within minutes to several hours after the administration of any form of exogenous progesterone, regardless of the route of administration. In addition to general patient data, the following information was collected and synthesized (when available): therapeutic justification for exogenous progesterone administration, product name and dose, route of administration, timing of the reaction, clinical presentation and WAO 2024 grading of the reaction [34], results of the allergy work-up (skin testing, in vitro testing, provocation tests), management of the acute episode and the successful strategy for enabling further necessary exogenous progesterone use.

The inclusion criteria for endogenous progesterone-induced anaphylaxis were: (1) a clinical presentation fulfilling the WAO 2020 anaphylaxis criteria, occurring consistently during the luteal phase of the menstrual cycle; and (2) either a positive skin test or provocation test with a progesterone product, and/or (3) complete resolution of symptoms under any form of progesterone-suppressive therapy. In addition, a positive challenge result following Gonadotropin/Luteinizing Hormone-Releasing Hormone (GnRH/LHRH) administration—used to increase endogenous progesterone levels—was also considered indicative. General patient data were collected, along with the following information, when available: the timing of symptom onset within the menstrual cycle, the clinical presentation and WAO 2024 grading of the reaction [34], history of prior exposure to exogenous progesterone, previous non-anaphylactic symptoms attributed to endogenous progesterone, results of the allergy work-up (skin testing, in vitro testing, provocation tests), management of the acute episode, ineffective therapies and the final successful long-term treatment approach. For this category, besides the age at diagnosis, we also recorded the age at symptom onset, when available. When the exact age of symptom onset was not mentioned but the duration of symptoms prior to diagnosis was reported, the age at symptom onset was estimated by subtracting that duration from the age at diagnosis. If symptoms had been present for less than 12 months, both ages were recorded as the same.

**Fig. 1:**
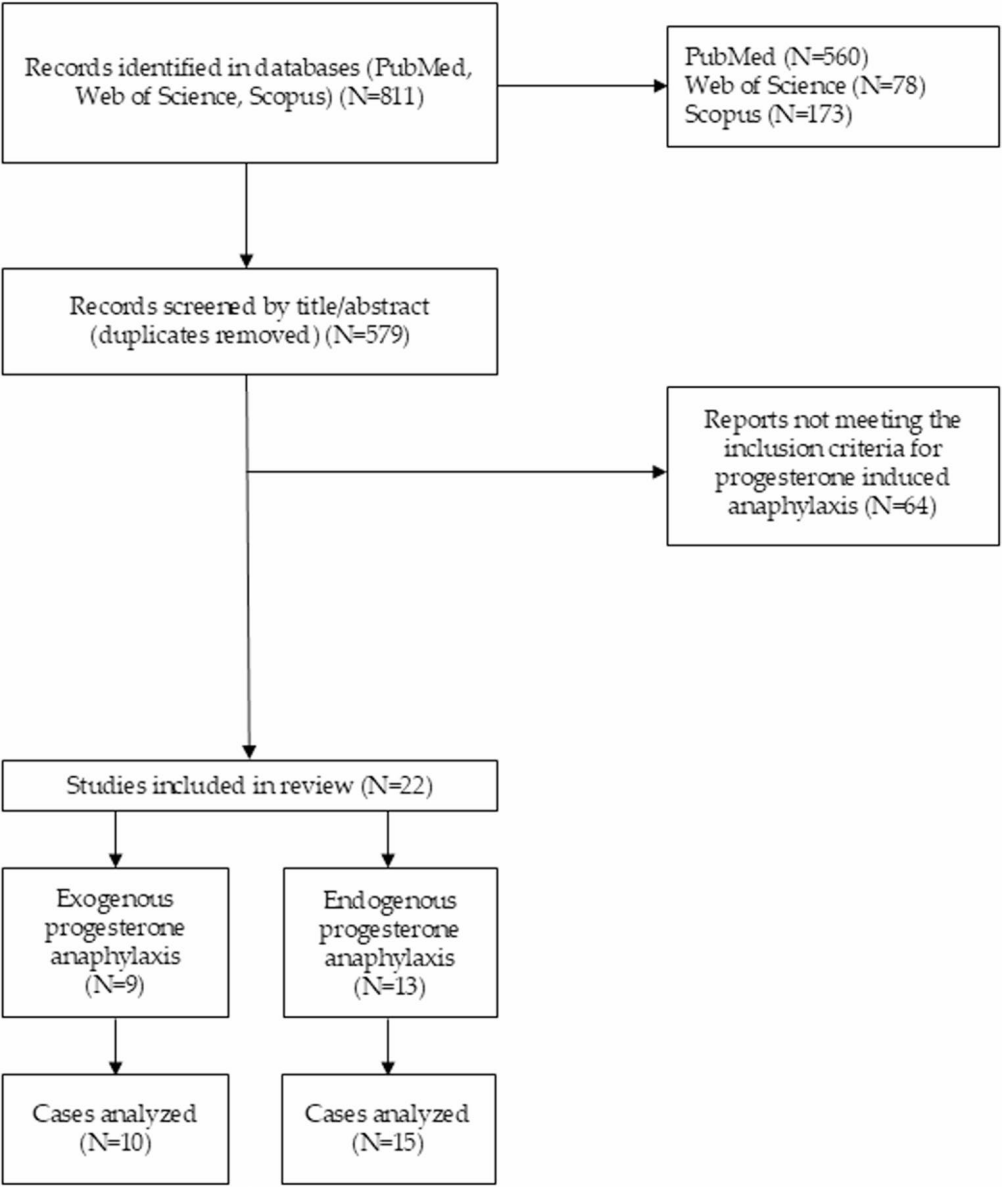
Flowchart of the literature search and selection process

The initial search across the three databases yielded 811 results (Fig. 1): 560 from PubMed, 78 from Web of Science and 173 from Scopus. After removing 232 duplicates, 579 articles remained for title/abstract screening to identify reports relevant to PH. The exclusion criteria were: (1) articles published in a language other than English; (2) articles not reporting a case related to PH; and (3) articles without sufficient information for data extraction and analysis. A total of 86 articles were identified as reporting cases related to PH. After applying the inclusion and exclusion criteria, 22 articles were included in the final analysis: 9 articles describing 10 cases of exogenous progesterone-induced anaphylaxis, and 13 articles describing 15 cases of endogenous progesterone-induced anaphylaxis.

## Results

### Exogenous Progesterone Anaphylaxis

The review included 9 articles related to exogenous progesterone anaphylaxis, corresponding to 10 patients (females) with 13 anaphylactic episodes described, with a median age of 30.5 years (range: 16–48), and parity ranging from multiparous to nulliparous, including one patient with primary infertility. A prior history of non-anaphylactic PH was reported in two patients. Both had experienced cyclic urticaria and angioedema before the index anaphylactic episode, suggesting prior endogenous progesterone sensitization. Exogenous progesterone was administered for various indications: luteal phase support during in vitro fertilization (2 patients), contraception (3 patients), control of menorrhagia (2 patients), management of menopausal symptoms (1 patient), menstrual cycle regulation (1 patient) and for one patient the reason was not specified. The progesterone formulations involved included medroxyprogesterone acetate (the most frequent trigger), natural micronized progesterone, progesterone gel, aqueous progesterone, and combined estrogen–progestin products in oral contraceptives. The most common route of administration was parenteral (intramuscular or unspecified injectable), followed by vaginal and oral routes. While most articles reported a single episode of anaphylaxis per patient, two patients experienced multiple episodes—two and three episodes, respectively. Symptoms typically occurred rapidly after administration, ranging from seconds to minutes. However, one case reported a delayed onset of symptoms 8–12 h after ingestion of an oral contraceptive, allowing for speculation regarding differences in absorption of the drug/ metabolization. According to the WAO 2024 anaphylaxis grading system, among the 13 reactions, six (46.2%) were classified as grade 3, another six (46.2%) as grade 4 and one (7.7%) as grade 5. In two cases involving infertility, anaphylaxis occurred during IVF cycles, and pregnancy was later achieved using a modified natural cycle without using exogenous progesterone or following desensitization protocols. Acute anaphylaxis management was reported in most cases: intramuscular epinephrine was administered in five reactions, often in combination with corticosteroids and H1/H2 antihistamines. In one severe case (grade 5), the patient required emergency intubation and mechanical ventilation in addition to epinephrine and systemic therapy. An allergy work-up was carried out in only four cases; two patients declined testing; only one progesterone provocation test was conducted, using a low dose of micronized progesterone, which yielded a positive result. Desensitization protocols were reported in three cases, and one patient required treatment for endogenous progesterone-induced symptoms following the anaphylactic reaction (Data summarized in Table 1).

**Table 1:** Summary of exogenous progesterone anaphylaxis cases

Author (Year)	Age	Prior PH history	Parity	Therapeutic justification for *P* adm.	Product (dose)	Route of adm.	Onset after adm.	Clinical presentation	Grade (WAO 2024)	Treatment	Skin tests	In Vitro tests	Provocation tests	Successful management
1. Brooks (1974) [35]	22	UNK	G3P2Ab0	Contraceptive	MPA (UNK)	Im	Seconds	U, facial AE, nausea, peripheral cyanosis	3	E, CS, AH	No	No	No	UNK
2. Zacest et al. (1982) [36]	23	UNK	UNK	UNK	MPA (UNK) 1st dose	Im	5 min	U, facial AE, photophobia, loss of consciousness, bronchospasm	4	AH	No	No	No	UNK
3. Rajapaksa (1993) [37]	46	UNK	UNK	Menorrhagia	MPA (UNK)	Im	Seconds	U, syncope, hTA, non-palpable pulse, bilateral rhonchi	4	CS, AH	No	No	No	UNK
4. Poole and Resenwasser (2004) [38]	28	Yes *	P1	Menopausal symptoms control	UNK	UNK	UNK	U, AE, wheezing, dyspnea	4	UNK	No	No	+ (U&AE) to micronized P (25 mg)	2-day P desensitization
5. Selo-Ojeme et al. (2004) [39]	40	UNK	P2	Contraceptive	MPA (150 mg)	Im	1st episode - minutes	Cardiorespiratory arrest	5	E, CS, AH	Refused	No	No	UNK
							2nd episode – seconds	Wheezing, cyanosis, sweat	4	E, CS, AH, intubation				
6. Prieto-Garcia et al. (2011) [40]	36 (Case 3)	No	Primary infertility	IVF	UNK	Inj.	UNK	U, AE, dyspnea, lightheadedness, nausea, abdominal colic, diarrhea, diaphoresis, presyncope	4	UNK	+ ID for P (0.5 mg/ml)	No	No	Intravaginal 10 steps P desensitization
	33 (Case 6)	Yes **	UNK	Menorrhagia	MPA (150 mg)	Im	UNK	U, dyspnea, cough, chest tightness	3	UNK	+ ID for P (5 mg/ml)	No	No	Oral 7 steps P desensitization
7. Lestishock et al. (2011) [41]	16	UNK	UNK	Contraceptive	MPA (150 mg) at 5th dose	Im	UNK	U, pallor, dyspnea, throat tightness	3	E, CS, AH H1 and H2	No ***	No	No	UNK
8. Bernstein et al. (2011) [42]	48	No	Nulliparous	Menstrual cycle regulation	Norethindrone/ ethinyl estradiol (0.5,0.75 and 1 mg/0.035 mg)	Oral	8–12 h	Labiofacial AE, bronchospasm, hTA – 5 episodes	4	UNK	- SPT, no ID****	+ sIgE and IgG to P, + LHR to P/5beta-pregnanediol, inhibited by anti-P Ab and mifepristone	No	LHRH
9. Gupta et al. (2018) [43]	27	UNK	G1P0Ab1	IVF	Natural micronized P (100 mg)	Inj.	2 h	Burning, pain at adm. site, breathlessness, fever	3	CS	No	No	No	Modified Natural Cycle without exogenous P – pregnancy successfully obtained
					Aqueous P (25 mg)	Inj.	UNK	Similar symptoms	3	UNK				
					P gel (300 mg)	Vaginal	UNK	Similar symptoms and vaginal blisters	3	UNK				

PH- progesterone hypersensitivity; P – progesterone; Adm. – administration; UNK- unknown; GPAb – Gravidity, pregnancy, abortion; MPA – medroxyprogesterone acetate; Im – intramuscular; U – urticaria; AE – angioedema; E – epinephrine; CS – corticosteroids; AH – antihistamine; hTA – hypotension; + – positive; IVF – in vitro fertilization; Inj – injectable; ID – intradermal; - – negative; sIgE – specific immunoglobulin E, IgG – immunoglobulin G; LHR - Leukocyte histamine Release ; Ab – antibody; LHRH - Luteinizing Hormone-Releasing Hormone

* Cyclical urticaria and angioedema since she was 13 years old.

** Hives, vulvar swelling and redness, dyspnea after oral contraceptives (0.15 mg desogestrel – 20 mg ethinyl E2).

***Refused tests to progesterone; skin tests to excipients: polyethylene glycol 3350, polysorbate 80, methylparaben, and propylparaben – equivocal result for polysorbate 80 for i.d. 1/100; positive for polyethylene glycol i.d. 1/10.

****Skin prick tests negative also to 5b-pregnanediol, estradiol, norethindrone, and Ortho-Novum-777; no intradermic tests

### Endogenous Progesterone Anaphylaxis

A total of 13 articles with 15 cases of anaphylaxis attributed to endogenous progesterone exposure were identified. The median age at diagnosis was 34 years (range: 12–48), and the onset of symptoms typically occurred in close relation to the luteal phase of the menstrual cycle, most commonly 1–3 days before menstruation. Among the 11 patients for whom age at symptom onset was reported, the average time from symptom onset to diagnosis was 3.27 ± 4.36 years (range 0–15). In several cases, the onset occurred during adolescence or early adulthood, suggesting that symptoms can appear relatively early after menarche. The patients had varying reproductive histories, including both nulliparous and multiparous cases. Prior exposure to exogenous progesterone was noted in five cases. Nine reactions (60%) were classified as grade 3 on the WAO 2024 anaphylaxis scale, and six (40%) as grade 4. The clinical presentation most frequently included urticaria, angioedema (facial, lingual, or laryngeal), respiratory symptoms (dyspnea, wheezing, chest tightness), and, in several cases, hypotension or syncope. A minority of cases also associated gastrointestinal symptoms or signs suggestive of capillary leakage (ascites, pleural effusion). Allergy work-up was performed in most cases. Skin testing yielded positive results in all 13 patients tested to progesterone, using various concentrations. In three cases, intradermal tests reproduced systemic symptoms similar to those observed during the index reaction. In one patient who underwent skin testing only to conjugated estrogens and diethylstilbestrol (with negative results) a provocation test was positive for medroxyprogesterone. For four other patients who had positive skin tests to medroxyprogesterone a provocation test was performed with LHRH in order to stimulate endogenous progesterone secretion. The LHRH challenge was positive in three out of four cases (75%). Notably, the patient with a negative LHRH challenge also failed to react to a control trial using a long-acting LHRH agonist. Management strategies varied. Acute treatment of reactions included epinephrine, corticosteroids, and antihistamines. Long-term preventive strategies ranged from antihistamines and leukotriene receptor antagonists to hormonal suppression using conjugated estrogens, LHRH long-acting agonists and oophorectomy. Omalizumab was used successfully in two patients (Data summarized in Table 2).

**Table 2:** Summary of endogenous progesterone anaphylaxis cases

Author (Year)	Age at diagnosis	Age at onset	Parity	Prior exogenous *P* adm.	Prior PH History (non-anaphylactic)	Symptoms	Severity grade WAO	Menstrual Cycle Timing	Acute Reaction Management	Skin Test (dose)	In vitro Test	Provocation test	Unsuccessful Control/ Preventive Treatments	Successful Preventive Treatment (No Recurrence)
1. Hart et al. (1977) [61]	48 (Case 5)	47	UNK	Yes, substance UNK	UNK	U, AE, laryngeal spasm	3	10 days before menstruation	E, CS	+ ID for P (dose UNK)	No	No	AH, CS, ACTH, oral and vaginal nystatin, desensitization injections	Conjugated estrogens
2. Meggs et al. (1984) [46]	36	33	G7P3Ab4	Yes, OCP and parenteral P	UNK	U, flushing, facial, lingual, laryngeal and labial AO, wheezing, bronchorrhea, hTA, tachycardia, substernal chest pressure, abdominal bloating	4	every 5 to 10 days	AH, theophylline, tracheal fenestration	+ ID for MPA**	No	+ to adm. of LHRH	OCP (induced attacks)	LHRH long-acting analogue; Oophorohysterectomy
3. Slater et al. (1987) [49]	24 (Case 1)	22	G0P0	UNK	UNK	Flushing, bloating, syncope	4	Throughout the menstrual cycle	UNK	+ ST for MPA (local flare) (dose UNK)	No	- to adm. of LHRH	LHRH long-acting agonist (even if ovarian hormone decreased and menstruation ceased)	UNK
	37 (Case 2)	31	G1P0Ab1	UNK	UNK	Flushing, bloating, nausea, U, laryngeal AE	3	Throughout the menstrual cycle	UNK	+ ST to MPA*** (dose UNK)	No	+ to adm. of LHRH (flushing, U, hTA)	No	LHRH long-acting agonist; Oophorectomy (incomplete remission)
	42 (Case 4)	27	G2P2	UNK	UNK	Flushing, facial AE, U, syncope	4	Throughout the menstrual cycle, premenstrual worsening	UNK	+ ST for MPA*** (dose UNK)	No	+ to adm. of LHRH (flushing, U, hTA)	No	LHRH long-acting agonist; Oophorectomy
4. Snyder et Krishnaswamy (2003) [62]	22	UNK	G1P1Ab0	Yes, OCP (norgestimate and ethinyl estradiol)	Yes (for 2 years)	Flushing, facial AE, U, respiratory distress, pleural effusion, ascites	3	3 days before menses, lasted 5 to 7 days	AH, CS	- ST for conjugated estrogens and diethylstilbestrol	No	+ to MPA (systemic reaction within 15 min of lowest dose)	UNK	LHRH agonists; Leuproreline; Hysterectomy with bilateral salpingo-oophorectomy
5. O’Rourke et al. (2004) [63]	37	UNK	G2P1Ab0	UNK	Yes (for 14 years) – diagnosed	Dyspnea, sensation of a lump in her throat, generalized swelling	3	During luteal phase of menstrual cycle	E, CS, AH	+ ID for P (5 mg/ml)	Antiprogesterone Ab	No	UNK	Hysterectomy and oophorectomy
6. Bemanian et al. (2007) [64]	18	13	UNK	UNK	UNK	U, AE, dyspnea, cough, noisy breathing;	3	2–3 days before menses; 2 days into menses	AH, salbutamol, CS (relief in respiratory symptoms)	+ ID for P (50 mg/ml)	No	No	UNK	Conjugated estrogen
7. Magen et al. (2012) [65]	24	22	G1P0Ab1	Yes, OCP	Yes	U, laryngeal AE, asthma, hTA	4	3–4 days before menses; 4 days into menses	UNK	+ ID for P (dose UNK)	No	No	AH	Conjugated estrogen; LHRH agonist (incomplete remission of skin symptoms)
8. Honda et al. (2014) [66]	21	21	UNK	UNK	UNK	U, severe respiratory distress	3	At the beginning of menses	CS	+ ID for P (10 mcg/mcL)	No	No	UNK	Spontaneous resolution of anaphylaxis after ST
9. De Rosa et al. (2015) [67]	41 (Case 3)	UNK	G5P3Ab2	UNK	Yes	Dysphagia, dyspnea, limbs AE	3	2 weeks every month (amenorrheic due to a hysterectomy 6 years earlier)	UNK	+ ID for P (dose UNK)	No	No	UNK	Synthetic P (norethindrone acetate)
10. Lin et al. (2017) [68]	48	48	UNK	UNK	Yes (for three years)	Tongue AE, stridor, dysphonia, respiratory distress	4	Within first days of menstruation	E, CS, AH	+ ST for MPA (dose UNK)	No	No	UNK	UNK
11. Heffler et al. (2017) [69]	12	12	UNK	No	Yes	U, labial and eyelid AE, dyspnea with chest tightness and wheezing, abdominal pain, nausea, diarrhea, hTA	4	2 days before the onset of menses	E, CS, AH	+ ID for P (0,05 mg/ml)	No	No	CS, AH	Omalizumab 300 mg sc monthly (6 months)
12. Lavery et al. (2019) [8]	16	14	UNK	Yes, OCP	No	Lip AE, dizziness, tingling and burning on face and lips, hands tingling and chest tightness	3	2–3 days before the menstrual cycle and continue into the cycle for 3–4 days*	UNK	No	Elevated sIgE to P	No	AH, LTRA; P desensitization	LHRH agonist, Omalizumab 300 mg subcutaneous injections and desensitization (to combined OCP)
13. Varghese et al. (2021) [60]	34	UNK	2 births	UNK	Yes	U, diffuse AE, headaches, dyspnea, laryngeal edema, nausea, vomiting, abdominal pain	3	1–2 days before the start of menstrual cycle	CS	+ ST to P (dose UNK)	No	No	AH, tricyclic antidepressants, calcineurin inhibitor, immunosuppressant therapy, PUVA therapy, OCP; Bilateral oophorectomy (resolved systemic, but not the skin symptoms)	Omalizumab

P – progesterone; Adm. – administration; PH- progesterone hypersensitivity; UNK- unknown; U – urticaria; AE – angioedema; E – epinephrine; CS – corticosteroids; + – positive; ID – intradermal; AH – antihistamine; ACTH – adrenocorticotropic hormone; GPAb – Gravidity, pregnancy, abortion; OCP – oral contraceptive pill; hTA – hypotension; MPA – medroxyprogesterone acetate; LHRH – Luteinizing Hormone – Releasing Hormone; Ab – antibody; LTRA - Leukotriene Receptor Antagonist; ST- skin test; PUVA – Psoralen + UVA

*1–2 days after the onset when oral contraceptive taken

**systemic reaction: diffuse urticaria, angioedema, bronchospasm after sequential administration of 10,20,40 mcg medroxyprogesterone

***systemic reaction, similar to their index reaction

## Discussion

This literature review highlights the potential severity of PH, which can present with a broad spectrum of clinical manifestations, ranging from mild symptoms to life-threatening anaphylaxis. It also underscores the heterogeneity in allergy work-up and the wide range of therapeutic strategies described in the literature, reflecting the overall complexity of this condition.

The earliest report of progesterone-induced anaphylaxis identified in our review dates back to 1974 [36], indicating that the condition has been recognized for several decades. A literature review published in 2003 specifically addressing progester-one-induced anaphylaxis reported only two cases attributed to exogenous progesterone and six to endogenous progesterone, highlighting the rarity of documented cases at the time and the exceptional nature of reactions to exogenous forms, likely due to their more limited use [37]. Since then, the number of published reports has increased. The present review identified more recent and better-characterized cases, which may reflect a rising incidence of the condition.

### Patients Profile and Clinical Characteristics

Although PH primarily affects women, and all patients in our review were female, one case has been reported in literature in a male patient presenting skin-limited symptoms while undergoing exogenous progesterone treatment (prescribed to stimulate appetite and weight gain in patients with cancer- or AIDS-related anorexia, regardless of gender) [44]. The median age of the patients in the reviewed cases was relatively young (34 years for exogenous and 30.5 years for endogenous, respectively), which aligns with previous findings suggesting that PH predominantly affects women in their peak reproductive years [15, 28, 45]. Regarding the pregnancy status of the patients at diagnosis, one patient, who experienced anaphylactic symptoms prior to conception, also presented recurrent anaphylaxis episodes specifically during the second and third trimesters of her pregnancy [46]. Furthermore, one parturient patient with a 14-year history of autoimmune progesterone dermatitis developed severe anaphylaxis during an emergency caesarean section. However, the reaction occurred 40 min after delivery, resulting in no harm to the newborn, who presented with Apgar scores of 8 and 10 [47]. Parity among patients was variable. While sensitization is often hypothesized to occur during periods of high endogenous progesterone levels, such as pregnancy [48], we also identified a case of anaphylaxis to endogenous progesterone in a nulliparous patient [49], suggesting that pregnancy is not a requirement for sensitization. Interestingly, in the exogenous progesterone group, one patient had a prior history suggestive of hypersensitivity to endogenous progesterone and despite high progesterone levels during pregnancy, this patient experienced complete symptom remission during that period [38]. This paradoxical improvement has also been reported by other authors in patients with non-anaphylactic PH [50–55]. One possible explanation is that pregnancy may act as a form of natural desensitization to progesterone, due to the gradual and sustained increase in endogenous hormone levels [48, 56]. Alternatively, this phenomenon could be related to a reduced maternal immune reactivity to progesterone during pregnancy [48, 56–59]. Hormonal transitions surrounding childbirth and breastfeeding may also influence symptom onset. In one case, symptoms began a few days postpartum [60], whereas in another, complete remission was observed during the breastfeeding period [49].

Previous exposure to exogenous progesterone was reported in 33.3% of patients with endogenous progesterone-induced anaphylaxis. This finding is consistent with variable results across other case series describing PH, highlighting that sensitization to endogenous progesterone can develop independently of prior exogenous exposure [45, 61, 70–72].

Several patients experienced non-anaphylactic symptoms suggestive of PH prior to the index anaphylactic reaction. In our review, such symptoms were reported in 7 out of 15 patients (46.6%) with endogenous progesterone-induced anaphylaxis, and in 2 out of 10 patients (20%) with exogenous progesterone-induced anaphylaxis, supporting the possibility of prior sensitization before the anaphylactic reaction (Tables 1 and 2). However, a review by Sandru et al. [73] which investigated PH reactions related to exogenous hormone administration during assisted reproductive techniques, found that most patients had no prior history of hypersensitivity, suggesting that sensitization to exogenous progesterone may occur even in the absence of previously recognized reactions to endogenous hormone. Following an anaphylactic reaction to a combined oral contraceptive, one patient from the exogenous group went on to develop persistent maculopapular dermatitis attributed to endogenous PH, persisting for more than 21 years [14]. This case provides evidence that exposure to exogenous progesterone may prime the individual’s response to endogenous progesterone despite molecular differences between the two. It also aligns with the mixed clinical phenotype proposed by Foer et al. [24].

The clinical manifestations described in our review highlight the potential severity of PH. One patient experienced grade 5 anaphylaxis, and 12 additional reactions were classified as grade 4, emphasizing the serious nature of this condition. Notably, in the case of the grade 5 anaphylaxis, the true trigger was initially misattributed to anesthesia. As a result, the patient was re-exposed to exogenous progesterone, which led to another severe reaction (grade 4) [39]. This case underscores the urgent need for increased awareness of this hormone (natural or synthetic) as a potential trigger of hypersensitivity reactions among healthcare professionals across specialties.

### Diagnosis

The diagnosis of PH is primarily based on a highly suggestive clinical history. In the articles included in this review, skin testing and provocation tests were used in some patients to support the diagnosis, while in others, a therapeutic response served as the main diagnostic tool. This therapeutic response refers to the administration of progesterone-suppressive therapy, followed by remission of symptoms due to the cessation of exposure to the triggering agent—progesterone. As noted by some authors, a therapeutic response may be sufficient to establish the diagnosis, and in cases with a strong clinical history, additional diagnostic procedures may not be necessary [53, 74, 75].

In the articles reviewed, skin testing yielded positive results in most patients tested. However, the reviewed cases illustrate marked heterogeneity in the allergy work-up, with skin testing performed only in a subset of patients and using widely variable concentrations, ranging from 0.05 mg/ml to 50 mg/ml. The guideline proposed by Broyles et al. [76], a key reference for standardizing cutaneous testing, recommended a maximum concentration of 50 mg/ml for prick testing and 5 mg/ml for intradermal testing. Higher concentrations (such as the 50 mg/ml solution for ID reported in some studies) produced positive reactions also in a control group of 10 healthy women, raising concerns about false positives [77]. The risk of false positive results can additionally be reduced by performing control tests with the diluent, particularly because progesterone is not water-soluble and is often formulated in oil- or ethanol-based solutions [78]. Of note, in one patient included in this review, previously positive skin test results became negative five months after discontinuation of omalizumab therapy [79].

The role of in vitro tests in the diagnosis of PH remains insufficiently defined. Although progesterone-specific IgE antibodies have been identified in some patients, and other tests such as leukocyte histamine release and interferon-gamma release assays have been used to support the diagnosis [15, 61, 80, 81], their clinical utility and accessibility still remains limited. In vitro investigations were reported in only 3 of the 25 cases included in this review, suggesting a modest role for such assays when a suggestive clinical history and symptom pattern are present [8, 14, 63, 74].

Provocation testing can be particularly useful in cases with an inconclusive clinical history. While direct provocation with exogenous progesterone appears to be the most straightforward approach, some practitioners have also employed LHRH administration as an indirect challenge method in patients with suspected endogenous progesterone-induced symptoms [74, 76]. In the studies included in our review, oral or intramuscular progesterone challenge confirmed the diagnosis in 2 out of 2 cases [38, 62] while LHRH challenge yielded positive results in 3 out of 4 patients [46, 49]. One patient with suspected endogenous PH had a negative LHRH challenge and did not respond to long-acting LHRH agonist therapy. Despite this, the patient’s skin test to medroxyprogesterone was positive, and the clinical history was highly suggestive of the diagnosis [82]. A possible explanation for the negative challenge result is that the progesterone levels induced by LHRH stimulation may not have reached the individual symptomatic threshold. This mechanism has also been proposed as an explanation in cases of false-negative results during exogenous progesterone challenge [53, 74]. Specific progesterone challenge protocols have been proposed by Chiarella et al. [56], aiming to standardize diagnostic procedures.

A significant limitation across the reviewed literature is the inconsistent reporting of comorbid mast cell disorders. Heffler et al. [69] were the only authors to explicitly state that masquerading conditions, specifically systemic mastocytosis, were considered and ruled out in their patient. The remaining studies did not document serum tryptase levels or specific screening for mast cell activation syndrome. This lack of screening is particularly concerning because the exclusion of mast cell disorders is clinically paramount to prevent misdiagnosis and inappropriate management. Distinguishing between a specific IgE-mediated allergy to progesterone and non-specific mast cell degranulation triggered by hormonal fluctuations ensures that patients receive targeted therapies. Crucially, an accurate differential diagnosis avoids unnecessary and irreversible surgical interventions, such as bilateral oophorectomy [60].

Finally, the role of excipients in exogenous progesterone formulations must also be considered, as they may significantly impact diagnostic accuracy. Sensitization to excipients was suspected in two patients included in the reviewed articles. One patient had a prior history of anaphylaxis to Alfathesin (alfaxalone/alfadolone acetate), a discontinued anesthetic agent. The authors hypothesized that the reaction may have been due to hypersensitivity to a solubilizing agent common to both Alfathesin and the medroxyprogesterone formulation [36]. In another case, the patient declined skin testing to progesterone itself but agreed to testing for the excipients present in the suspected preparation, including polyethylene glycol 3350, polysorbate 80, methylparaben, and propylparaben. A positive intradermal reaction to polyethylene glycol 3350 (at a 1:10 dilution) was observed. Interestingly, the same substance was later tolerated when administered orally, suggesting that the route of exposure may influence the clinical expression of hypersensitivity [41].

### Management

The acute management of anaphylaxis should follow established therapeutic guidelines [33]. Yet, in our review, epinephrine was administered in only 4 out of 15 cases (26.6%) involving endogenous progesterone-induced reactions, and in 4 out of 13 episodes (30.7%) attributed to exogenous progesterone. This low rate of epinephrine use may reflect limited awareness among healthcare professionals regarding progesterone as a potential trigger of anaphylaxis. In consequence, the clinical suspicion for anaphylaxis may be delayed or misdirected, especially when the hormonal exposure is not initially perceived as allergenic. An illustrative case is the one reported by Selo-Ojeme et al. [30], in which the initial reaction was misattributed to anesthesia. As a result, progesterone was subsequently re-administered, leading to a second anaphylactic episode [39].

Long-term management should be tailored to each patient’s therapeutic goals. For some patients, symptom control may be the primary objective, while others may desire to pursue pregnancy, with or without assisted reproductive techniques such as IVF.

Across the cases included in this review, symptom control was tempted unsuccessfully using antihistamines, corticosteroids and leukotriene receptor antagonists. Suppression of endogenous progesterone secretion was approached through various strategies, including long-acting GnRH agonists (LHRH analogues), conjugated estrogens, and oophorectomy. Additionally, omalizumab was employed in selected cases, particularly when conventional treatments failed or when hormonal suppression could no longer be prolonged [8, 60, 79]. Although oophorectomy is viewed as a definitive approach, complete symptom resolution was achieved in four out of six patients [46, 62, 63, 82], while two presented only partial improvement [49, 60]. Notably, in one patient, symptom control was achieved through the administration of synthetic progesterone [67], whereas in others, exposure to exogenous progesterone as part of combined oral contraceptives triggered hypersensitivity reactions [60, 83].

Regarding strategies for symptom control induced by exogenous progesterone, changing the route of administration (oral, vaginal, or injectable) and, consequently, the excipients, was unsuccessful when the active product remained the same. Specifically, Gupta et al. [43] reported a patient who developed hypersensitivity reactions to natural micronized progesterone regardless of its formulation, reacting sequentially to aqueous intramuscular injections, vaginal gel and oral capsules. Furthermore, the rotation between different classes of synthetic progestins and natural progesterone has also proven ineffective. Poole and Rosenwasser [38] described a patient who exhibited pan-sensitivity to 19-nortestosterone derivatives (Norethindrone), 17-hydroxyprogesterone derivatives (Medroxyprogesterone Acetate), spironolactone analogues (Drospirenone) and natural micronized progesterone. This suggests that in cases of total cross-reactivity, switching the chemical class of the progestogen may not prevent anaphylaxis.

However, in case of exogenous induced symptoms, if the hormonal supplementation is mandatory, a desensitization protocol is the only option. Among the cases included in our review, this approach was successfully applied in one patient, who subsequently carried a pregnancy to term and gave birth to twins [40]. The protocols vary in terms of number of steps, duration and administration route (oral, intravaginal), but also in goal, the desensitization being mandatory not only for IVF procedures but also for other conditions that need hormonal supplementation such as uterine bleeding [38, 72]. Additionally, desensitization protocols using intramuscular progesterone have been proposed, with both rapid and slow escalation approaches reported [24, 75, 84, 85].

Desensitization protocols have also been applied in cases of endogenous progesterone-induced anaphylaxis, particularly when other therapeutic strategies have failed. In the present review, two patients were identified as having unsuccessful desensitization attempts; however, details regarding the protocols used were not provided [8, 61]. For one of these patients, a successful outcome was eventually achieved through the addition of omalizumab to the desensitization protocol involving a combined oral contraceptive [8].

A noteworthy observation was made in one case, where anaphylactic symptoms resolved spontaneously following skin testing, but the manifestations shifted to a fixed drug eruption at the injection site. This suggests a possible modulation of the immune response after allergy evaluation [66].

Other treatment options for PH, though not yet reported in patients with anaphylaxis, have shown promising results in milder forms of the condition. These include danazol [70, 74, 86, 87], Janus kinase inhibitor [88], azathioprine [89, 90] and dapsone [91].

**Fig. 2:**
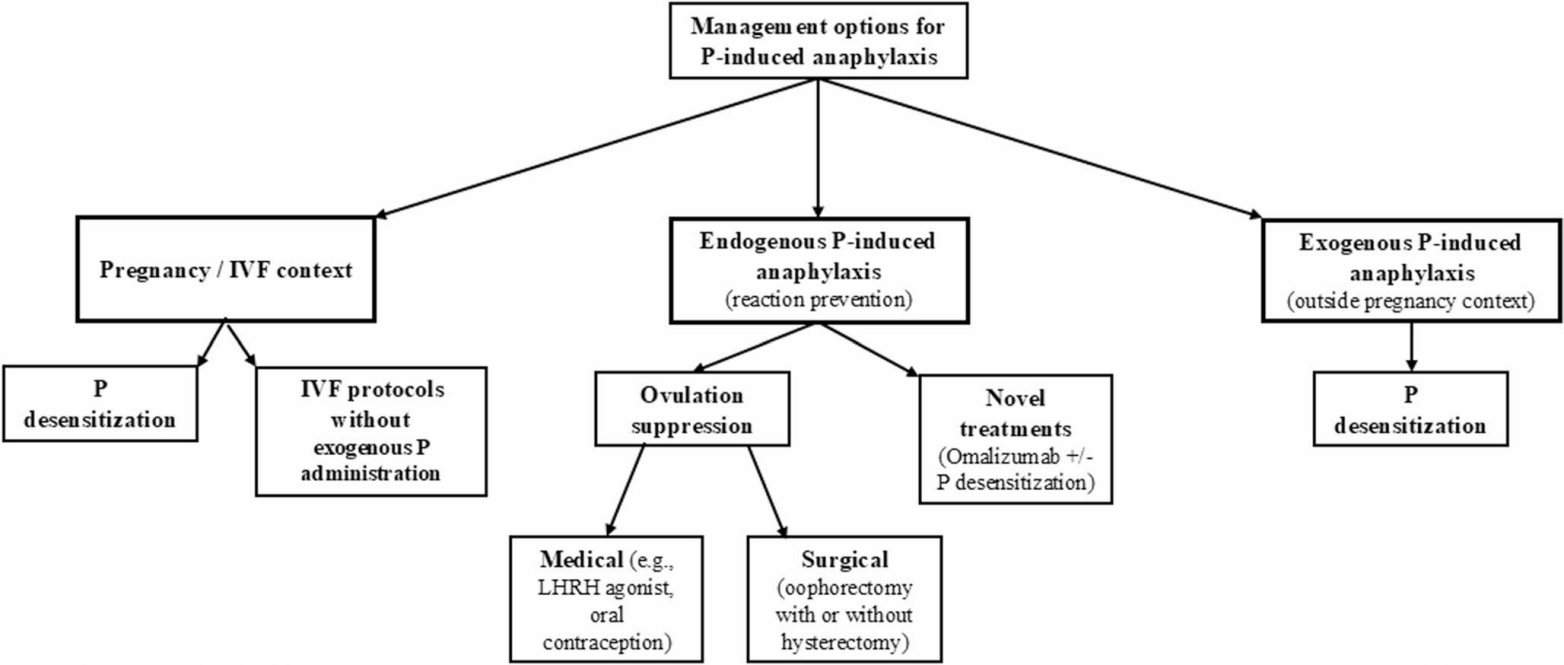
Proposed management algorithm for P-induced anaphylaxis

As a corollary of the reviewed cases and reported outcomes, we elaborated a decision algorithm for progesterone-induced anaphylaxis (Fig. 2). In the context of pregnancy or assisted reproduction (IVF), management options include intramuscular progesterone desensitization or, when feasible, a modified natural cycle approach without the need for exogenous progesterone. When the goal is to control symptoms induced by endogenous progesterone, treatments with antihistamines, leukotriene receptor antagonists, or corticosteroids did not appear effective in the reviewed reports. In such cases, the most widely adopted strategy was ovulation suppression, achieved either medically—with long-acting LHRH agonists or oral contraceptives (estrogen-only, or combined formulations if low-dose progesterone was tolerated)—or surgically, through oophorectomy with or without hysterectomy. The choice of strategy should take into account the patient’s age and reproductive goals. Omalizumab, alone or in combination with progesterone desensitization, may represent an alternative when ovulation suppression is not desirable. The choice between medical and surgical management strategies indeed requires careful consideration of the balance between risks, benefits, and reversibility and should be made in close consultation with the treating specialist. Finally, in cases of exogenous progesterone–induced anaphylaxis, where administration remains essential even outside the pregnancy context (e.g., for menorrhagia), progesterone desensitization is the only viable option. Overall, management requires an individualized approach tailored to the patient’s clinical context and must be decided in close collaboration with a gynecologist or reproductive medicine specialist.

### Limitations

The key limitation relates to the way progesterone hypersensitivity was diagnosed in the reviewed cases. In most reported cases, diagnosis was primarily based on clinical history and the temporal relationship between symptoms onset and progesterone exposure, with limited use of confirmatory allergy testing. Moreover, the retrospective assessment of severity from case reports has inherent limitations. Not all publications provided sufficient clinical detail to confidently assign a WAO grade, which may have led to under- or overestimation of severity in some instances.

In the case of exogenous progesterone-induced reactions, we included all reports in which the clinical presentation was consistent with anaphylaxis and the timing was compatible with progesterone administration, without requiring a confirmed allergy work-up. This approach allowed for a broader inclusion of cases and a more comprehensive overview of possible progesterone-induced reactions. Only three of the included patients underwent a confirmatory skin or provocation test [38, 72]. This limitation is partly explained by the known reluctance of patients (and probably clinicians) to perform allergy workups involving re-exposure to progesterone after an anaphylactic event. In contrast, for endogenous progesterone-induced anaphylaxis, we applied an additional criterion, in concordance with the diagnosis criteria proposed by Chiarella et al. [56]: besides a compatible timing (symptom onset during the luteal phase), we required either a positive allergy test or a clinical response to ovulation suppression agents. Additionally, a recent publication proposed a diagnostic algorithm in which, for cases involving anaphylaxis, the authors recommended the use of in vitro tests only, if available [81].

## Conclusions

PH remains an underdiagnosed condition with clinical implications for women of reproductive age. This narrative review provides an overview of the reported cases of progesterone-induced anaphylaxis, highlighting the potential for severe reactions. Failure to recognize this condition may place patients at risk, especially when progesterone is required in the context of assisted reproductive techniques or hormonal therapies. Beyond providing clinical guidance, our work also aims to serve as a useful resource by summarizing the available allergological work-up and reporting management strategies that have been successfully applied.

Greater awareness is essential among healthcare providers, especially gynecologists, allergists, and fertility specialists—in order to ensure accurate diagnosis and prompt management. Furthermore, many aspects of pathophysiology remain poorly understood, and future research is essential to elucidate the mechanisms of sensitization and to develop standardized allergy work-up and therapeutic protocols.

## Data Availability

No datasets were generated or analysed during the current study.

## Key References

- Chiarella, S.E.; Buchheit, K.M.; Foer, D. Progestogen Hypersensitivity. J. Allergy Clin. Immunol. Pract. 2023, 11, 3606+, doi:10.1016/j.jaip.2023.07.050.
  - ○ This authoritative review redefines progesterone-related reactions as progestogen hypersensitivity and proposes pragmatic diagnostic criteria incorporating clinical timing, allergy testing, and response to ovulation-suppressive therapies. It also discusses pregnancy as a potential state of natural progesterone desensitization, relevant to endogenous progesterone–induced anaphylaxis.
- Sandru, F.; Dumitrascu, M.C.; Petca, A.; Petca, R.-C.; Roman, A.-M. Progesterone Hypersensitivity in Assisted Reproductive Technologies: Implications for Safety and Efficacy. J. Pers. Med. 2024, 14, doi:10.3390/jpm14010079.
  - ○ This review addresses progesterone hypersensitivity related to exogenous hormone exposure during assisted reproductive techniques and shows that most patients had no prior history of hypersensitivity. These findings support the concept of de novo sensitization to exogenous progesterone in fertility treatments.
- Bello, C.D.A.; Guzman, O.P.G.; Coello, C.V.M.; Gutierrez, M.I.R.; Vazquez, M.I.C. Diagnostic Tests for Progestogen Hypersensitivity. Front. ALLERGY 2024, 5, doi:10.3389/falgy.2024.1384140.
  - ○ This review emphasizes the absence of standardized diagnostic tests and the limited performance of progesterone skin testing. It proposes a diagnostic algorithm favoring in vitro testing in patients with a history of anaphylaxis to minimize the risk of progesterone re-exposure.
